# Impact of psychiatric comorbidities in psoriasis, hidradenitis suppurativa and atopic dermatitis: The importance of a psychodermatological approach

**DOI:** 10.1111/exd.14563

**Published:** 2022-03-31

**Authors:** Michela Iannone, Agata Janowska, Salvatore Panduri, Riccardo Morganti, Giulia Davini, Marco Romanelli, Valentina Dini

**Affiliations:** ^1^ 9310 Department of Dermatology University of Pisa Pisa Italy; ^2^ 9310 Department of Clinical and Experimental Medicine Section of Statistics University of Pisa Pisa Italy

**Keywords:** atopic dermatitis, hidradenitis suppurativa, inflammatory skin disease, psoriasis, psychiatric comorbidity, psychodermatological approach, psychological burden

## Abstract

**Background:**

There is a strong interaction between the immunological and nervous system in the skin. Lesions that are physically disfiguring and chronically relapsing have a high impact on quality of life (QoL) and can result in the emergence of psychiatric disorders. The literature data confirm a higher prevalence of psychiatric disorders in patients with psoriasis, hidradenitis suppurativa (HS) and atopic dermatitis (AD), but such data are compromised by low‐quality evidence due to methodological heterogeneity.

**Objectives:**

The primary aim was to analyse the prevalence of psychiatric comorbidities in a group of psoriasis, AD and HS patients compared with a control group. The secondary aims were to evaluate the impact of psychiatric comorbidities on the disease development, severity, flare‐ups and QoL.

**Methods:**

A total of 59 cases and 64 controls were included.

**Results:**

Generalized anxiety disorder and depressive disorder with anxious distress were found to be risk factors for AD. Age, smoking and substance‐related disorder showed a specific association with HS. Major depressive disorder showed a specific association with dermatology life quality index (DLQI) and all the above disease flare‐ups.

**Conclusions:**

Atopic dermatitis, psoriasis and HS are associated with psychiatric disorders. A psychodermatological approach improves outcomes in terms of QoL, disease flare‐ups and long‐term management.

## 1 BACKGROUND

There is a strong interaction between the immunological and nervous system in the skin. This relationship is investigated by psychoneuroimmunology and can be divided into three different types: (1) psychophysiological disorders; (2) primitive psychiatric disorders and (3) secondary psychiatric disorders.^1^, ^2^ Anxiety disorder is the most common primary psychiatric disorder in patients with chronic inflammatory skin diseases, with a prevalence of 17.2%.^3^ Secondary psychiatric disorders can alter the quality of life (QoL) of patients, with reactive depression, anxiety, social isolation and suicidal ideation. In chronic inflammatory skin diseases, particularly psoriasis, hidradenitis suppurativa (HS) and atopic dermatitis (AD), patients often have physically disfiguring lesions associated with itching, sleep disturbance, excoriation or bleeding with a chronic relapsing course characterized by unpredictable flare‐ups and difficult triggers to prevent. Such pathologies require an adaptation to living with the disease, which can generate psychiatric disorders.^3^, ^4^

Psoriasis is a chronic inflammatory skin disease characterized by an immune dysfunction of the Th1/Th17 axis.^5^ The first studies on the association between psoriasis and anxiety, depression and suicidal ideation were published at the end of the 1970s.^6^, ^7^ A systematic review of the literature from 1990 to 2015 confirmed the association of psoriasis with anxiety and depression. It also highlighted other comorbidities, such as eating disorders, psychotic disorders, personality disorders, sexual dysfunction, sleep disturbances, somatoform disorders and substance abuse.^8^ Another comprehensive qualitative review on psoriasis and psychiatric comorbidities since 2005 confirmed the findings given in^8^ but highlighted the poor quality of the data due to methodological heterogeneity (different mental health questionnaires, patients' self‐reported online surveys and several confounders). Although no reliable biomarkers exist for psychiatric disorders, clinical examination combined with structured interviews and diagnostic tools relying on the Diagnostic and Statistical Manual of Mental Disorders (DSM‐5) criteria allows to make a precise diagnosis.^9^

Atopic dermatitis (AD) is a chronically relapsing skin disease. Clinical hallmarks are intense itching and eczematous lesions, with a significant impact on daily life.^10^ The association of AD with depression, anxiety and suicidal ideation was confirmed by a systematic review and meta‐analysis published in 2018. The main limitation of this latter study was the definition of depression and anxiety based on self‐reported symptoms and not on clinical psychiatric diagnoses. Moreover, few studies have examined the disease's severity.^11^

Hidradenitis suppurativa (HS) is a chronic skin disease, characterized by recurrent and painful nodules, abscesses and sinus tracts involving the hair follicle.^12^ Living with HS indefinitely has a high physical and social impact: the presence of draining malodorous lesions in sensitive areas of the body has been described by patients as "miserable and horrible”.^13^ The first study on the association between HS and psychiatric disorders was published in 2012: depression, anxiety, bipolar disorder, psychoses, schizophrenia, substance abuse disorders and suicidal ideation are more common in HS patients than healthy individuals.^14^, ^15^ Patients affected by HS also have an alexithymic personality trait, which leads to the frequent development of generalized anxiety disorder or depression.^16^ A systematic review and meta‐analysis of the literature regarding the association between HS and psychiatric comorbidities revealed poor methodological quality and different clinical criteria for defining psychiatric comorbidities in published studies. These associations limit the prevalence of the comorbidities reported.^17^

## 2 QUESTIONS ADDRESSED

The primary aim was to analyse the prevalence of psychiatric comorbidities in a skin disease population compared with a control group. The secondary aims were to evaluate the impact of psychiatric comorbidities on disease development, severity, flare‐ups and QoL.

## 3 EXPERIMENTAL DESIGN

This retrospective study was performed in accordance with the Declaration of Helsinki and was approved by the Institutional Ethics Committee. We recruited adult patients diagnosed with psoriasis (20), HS (23) and AD (16) from February 2016 to October 2020. Patients were divided into two groups: cases with chronic inflammatory skin diseases and psychiatric comorbidities (depressive disorder with anxious distress; major depressive disorder; generalized anxiety disorder; substance‐related disorder; bipolar disorder; diagnosed and treated by the psychiatrist in accordance with DSM‐5 criteria) and controls with AD, HS or psoriasis without psychiatric comorbidities. Controls were matched for sex, age and disease severity to minimize the study bias.

Individual patient data were collected in open‐access databases with the following structure: patient initials, sex, age, disease severity (Psoriasis Area Severity Index (PASI) or Eczema Area Severity Index (EASI) or mSartorius), mean flare‐ups in the past 6 months, DLQI, current therapies and psychiatric comorbidities. The data were then entered into a single Excel file and subjected to descriptive and multivariate statistical analysis using SPSS v.25 software. Statistical significance was assessed in terms of *p*‐value and odds ratio.

## 4 RESULTS

A total of 59 cases and 64 controls were included. Table 1 reports the characteristics of the population.

**TABLE 1:** Characteristics of population. Statistics: frequency (%) or mean (SD)

Variables	Statistics
Age	46 (19)
Gender	
M	60 (48.8)
F	63 (51.2)
Clinical phenotype	
Atopic dermatitis	32(26)
Hidradenitis	45 (36.6)
Psoriasis	46 (37.4)
Smoke habits	
Yes	67 (54.5)
Not	56 (45.5)
Depressive disorder with anxious distress	
Yes	12 (9.8)
Not	111 (90.2)
Major depressive disorder	
Yes	20 (16.3)
Not	103 (83.7)
Generalized anxiety disorder	
Yes	16 (13)
Not	107 (87)
Substance‐related disorder	
Yes	7 (5.7)
Not	116 (94.3)
Bipolar disorder	
Yes	11 (8.9)
Not	112 (91.1)
Concomitant medications	
Biological treatment	58 (47.2)
Phototherapy	5 (4.1)
Systemic treatment	26 (21.1)
Topical treatment	34 (27.6)

The prevalence of psychiatric comorbidities was 13.90% in patients with psoriasis, 14.90% in HS patients and 19.8% in DA patients.

Table 2 shows the results of the multivariate binary logistic regression analysis performed using a stepwise method to assess the impact of psychiatric comorbidities, gender, age and smoking on dependent variables such as inflammatory skin diseases (DA, HS and psoriasis), objective disease score (PASI for psoriasis/EASI for atopic dermatitis/mSartorius for HS), DLQI and on disease flare‐ups. Objective disease scores were dichotomized into mild (<10) and moderate‐to‐severe (≥10); DLQI was dichotomized into <12, ≥12 and disease flare into <3, ≥3 and then subjected to binary logistic regression.

**TABLE 2:** Results of multivariate binary logistic regression analysis with stepwise method (statistically significant data highlighted in yellow)

		CR	*p*‐value	OR	IC 95% per OR	
					Inferior	Superior
Dependent variable: atopic dermatitis (no, yes)						
Phase 1	Smoke	−1.933	0.000	0.145	0.056	0.372
	Costant	−0.215	0.424	0.806		
Phase 2	Smoke	−2.051	0.000	0.129	0.047	0.351
	Depressive_Disorder_Anxious_Distress	1.851	0.011	6.367	1.540	26.329
	Costant	−0.394	0.163	0.674		
Phase 3	Smoke	−2.283	0.000	0.102	0.034	0.304
	Depressive_Disorder_Anxious_Distress	2.110	0.007	8.248	1.784	38.132
	Generalized_Anxiety_Disorder	1.655	0.019	5.233	1.317	20.791
	Costant	−0.576	0.055	0.562		
Dependent variable: Psoriasis (no, yes)						
Phase 1	Age	0.045	0.000	1.047	1.023	1.071
	Costant	−2.674	0.000	0.069		
Dependent variable: Hidradenitis (no, yes)						
Phase 1	Smoke	1.802	0.000	6.065	2.567	14.328
	Costant	−1.653	0.000	0.191		
Phase 2	Age	−0.060	0.000	0.942	0.914	0.971
	Smoke	2.551	0.000	12.821	4.258	38.608
	Costant	0.450	0.442	1.569		
Phase 3	Age	−0.060	0.000	0.942	0.914	0.970
	Smoke	2.439	0.000	11.460	3.826	34.324
	Substance_Related_Disorder	2.969	0.072	19.471	0.767	494.608
	Costant	0.431	0.467	1.540		
Dependent variable: Dermatology Life quality INDEX (<12, ≥12)						
Phase 1	Major_Depressive_Disorder	2.412	0.002	11.152	2.460	50.566
	Costant	−0.214	0.279	0.807		
Phase 2	Smoke	1.075	0.007	2.931	1.334	6.438
	Major_Depressive_Disorder	2.017	0.011	7.519	1.602	35.297
	Costant	−0.741	0.010	0.477		
Dependent variable: disease flares (<3, ≥3)						
Phase 1	Major_Depressive_Disorder	1.637	0.013	5.142	1.420	18.621
	Costant	0.097	0.622	1.102		
Dependent variable: objective disease scores (mild<10, moderate‐to‐severe≥10)						
Phase 1	Smoke	1.289	0.001	3.630	1.704	7.732
	Costant	−0.288	0.287	0.750		

All the comorbidities such as smoking, age, gender and psychiatric comorbidities were defined as independent variables and coded as follows: 0 = no; 1 = yes. Dermatologic diseases (AD, HS and psoriasis) were also coded in the same manner.

Generalized anxiety disorder and depressive disorder with anxious distress were found to be risk factors for AD development. None of the above variables showed a specific association with psoriasis. Age, smoking and substance‐related disorder showed a specific association with HS development. Major depressive disorder showed a specific association with DLQI and disease flare‐ups. None of the above independent variables impacted on objective disease scores. The statistical significance of the impacting variables is summarized in Table 3.

**TABLE 3:** Statistical significance of independent variables impacting on dermatologic diseases

Variables	Atopic dermatits Not	Atopic dermatitis Yes	*p*‐value
Smoke			
Not	31	25	<0.0001
Yes	60	7	
Depressive disorder with anxious distress			
Not	86	25	0.007
Yes	5	7	
Generalized anxiety disorder			
Not	82	25	0.083
Yes	9	7	

Variables	Hidradenitis Not	Hidradenitis Yes	*p*‐value
Age	50 (19)	38 (15)	0.001
Smoke			
Not	47	9	<0.0001
Yes	31	36	
Substance‐related disorder			
Not	77	39	0.005
Yes	1	6	

*Variables*	DLQI < 12	DLQI > 12	*p*‐value
Smoke			
Not	37	19	0.0002
Yes	22	45	
Major depressive disorder			
Not	57	46	0.0002
Yes	2	18	

Variables	Flares < 3	Flares > 3	*p*‐value
Major depressive disorder			
Not	49	54	0.007
Yes	3	17	

## 5 DISCUSSION

Many studies on psoriasis, HS and AD are extremely heterogeneous and use self‐administered questionnaires for the diagnosis of psychiatric comorbidities (such as Hospital Anxiety and Depression Scale, General Health Questionnaire‐28, Hamilton Anxiety Rating Scale, Epidemiological Studies Depression Scale, etc.). Self‐administered questionnaires highlight the symptoms of the anxiety or depressive spectrum but are not diagnostic with a high risk of false positives, poor‐quality evidence and an overestimation of the prevalence of psychiatric comorbidities.^9^, ^11^, ^17^

Our results showing the non‐impact of psychiatric comorbidities on psoriasis differ from those in the literature, which show an increased risk of depression, anxiety and suicidality in psoriasis patients.^18^, ^19^ This could be related to the strong bias in the interpretation of the published data for the different clinical criteria and the utilization of self‐administered questionnaires for the diagnosis of psychiatric comorbidities. The use of a common methodology and more studies on patients with specialist‐confirmed psychiatric diagnoses will help clarify the bias between psychological/psychiatric spectrum symptoms and true psychiatric comorbidities.

Our results on HS patients are in line with those reported by a Danish case‐control study that found no significant differences in the prevalence of depression in the two analysed groups.^14^ Other studies confirmed an association between HS, anxiety and depression; however, the severity of the disease was not considered in the selection of patients. It cannot be excluded that the selected patients all had severe forms of the disease and that the results are a consequence of the poor prognosis. Since the studies were cross‐sectional, the cause‐effect relationship cannot be defined.^20^, ^21^

Results on substance‐related disorder in HS patients should be contextualized in terms of the contrasting literature data: according to some authors, the association is significant, whereas for others, the concurrence of other psychiatric comorbidities reduces the significance of the association. In our patients, the association with a psychiatric comorbidity was found only in one case. Further studies are needed to clarify these data.

Our results on AD confirm an association with generalized anxiety disorder and major depressive disorder. The association with generalized anxiety disorder is in accordance with the literature data that define this disorder as "epiphenomenon indicative of hyperactivity of the hypothalamic‐pituitary‐adrenal axis”.^3^, ^22^ The chronic hyperactivation of this axis induces an increase in the diurnal cortisol secretion, mast cells and eosinophil activation and the isotypic switch of B lymphocytes into IgE‐secreting cells promoting a Th2‐type inflammation.^1^, ^4^, ^23^, ^24^, ^25^ Further studies are needed to verify whether this finding is repeatable for other atopic march comorbidities.

In both psoriasis and HS and AD patients, the impact of major depressive disorder on median DLQI values proved to be statistically significant, while there was no impact on disease objectivity. This finding has been reported in patients with psoriasis, HS and AD, but also with other chronic inflammatory diseases.^26^, ^27^, ^28^, ^29^ In order to improve the outcomes, it is, therefore, essential to evaluate the patient's subjective perception of the disease and then to correct and prevent depressive symptoms through group therapy, adjuvant training or the analysis of predisposing factors.^30^

Major depressive disorder also impacts on flare‐ups of the chronic inflammatory diseases under study. The concept of flares in the literature is highly variable. We considered a flare of HS as “an acute development of at least one inflammatory lesion or the recurrence of a previous lesion”; a flare of psoriasis/AD as "a worsening of PASI/EASI by at least 25% from baseline."

The association between major depressive disorder and flare‐ups of chronic inflammatory diseases is not reported in the literature. The neuroinflammatory hypothesis is one of the possible explanations for this association. More specifically, the excess of proinflammatory cytokines involved in the flare‐ups reduces the neurotrophic support with altered uptake/release of glutamate, consequent cytotoxicity and loss of glial elements, in line with the neuropathological findings that characterize depressive disorders.^31^

Our results showed that psychiatric comorbidities had no impact on disease severity, assessed in terms of PASI, EASI and mSartorius. There are little data on this topic in the literature. A possible association between disease severity and depression was reported in a German paper on psoriasis, in which, however, severity was estimated according to the number of medical visits in relation to the disease made by the patient.^32^ For AD, several studies associate psychological distress with major disease severity but no correlation with psychiatric comorbidities is described.^33^

The limitations of our study are the monocentricity and the small sample, which restrict the generalization of data. The strengths include the selection of patients with psychiatric comorbidities according to DSM‐5 criteria (diagnosed and treated by the psychiatrist), the assessment of dermatological diagnoses in a third‐level hospital and the selection of the case‐control population based on age, sex and disease severity in order to minimize bias. In contrast with other studies on large registry‐based populations, this enabled us to evaluate the absence of an association between disease severity and psychiatric comorbidities, that is data not currently reported in the literature.

## 6 CONCLUSIONS AND PERSPECTIVES

The closest interaction between the nervous and immunological systems in the skin occurs through a complex mix of psychosocial factors and psychiatric disorders. Chronic inflammatory skin diseases, particularly AD, psoriasis and HS, can cause psychiatric disorders. In order to improve the outcomes in terms of QoL, disease flare‐ups and long‐term management, we recommend that the medical therapy should be combined with a psychodermatological approach through strategies ranging from psychotherapy to pharmacotherapy, in collaboration with the specialist psychologist/psychiatrist.

The development of a common methodology with more studies on dermatological patients with specialist‐confirmed psychiatric comorbidities is needed in order to clarify the bias between psychiatric spectrum symptoms and psychiatric comorbidities in chronic inflammatory skin diseases. Prospective studies and clinical trials are needed to clarify the cause‐and‐effect relationships and to determine whether monitoring psychiatric comorbidities can improve patient perception of illness and reducing disease flares.

## CONFLICT OF INTEREST

The research reported in this article has not received external funding. No competing financial interests exist. The authors have no conflicts of interest to declare.

## AUTHOR CONTRIBUTIONS

Michela Iannone, Salvatore Panduri, Riccardo Morganti, Giulia Davini and Agata Janowska contributed to the conception and design, acquisition of data, analysis and interpretation of data. Valentina Dini and Marco Romanelli were involved in drafting the manuscript and revised it critically for important intellectual content.

## INFORMED CONSENT

The submitting author has the written consent from all authors to submit the manuscript. All authors have participated sufficiently in the work to take public responsibility for appropriate portions of the content and accept complete responsibility for the contents of the manuscript. The authors have agreed to be accountable for all aspects of the work in ensuring that questions related to the accuracy or integrity of any part of the work are appropriately investigated and resolved. The authors warrant that the article is original, is not under consideration by another journal and has not been previously published.
